# CO-Sprout—A Pilot Double-Blinded Placebo-Controlled Randomised Trial of Broccoli Sprout Powder Supplementation for Pregnant Women with COVID-19 on the Duration of COVID-19-Associated Symptoms: Study Protocol

**DOI:** 10.3390/nu15183980

**Published:** 2023-09-14

**Authors:** Neville J. Fields, Kirsten R. Palmer, Daniel L. Rolnik, Jennifer Yo, Marcel F. Nold, Michelle L. Giles, Sushena Krishnaswamy, Ary Serpa Neto, Ryan J. Hodges, Sarah A. Marshall

**Affiliations:** 1 The Ritchie Centre, Department of Obstetrics and Gynaecology, School of Clinical Sciences, Monash University, Melbourne 3168, Australiadaniel.rolnik@monash.edu (D.L.R.); marcel.nold@monash.edu (M.F.N.); michelle.giles@monash.edu (M.L.G.); ryan.hodges@monash.edu (R.J.H.); sarah.marshall@monash.edu (S.A.M.); 2 Monash Health, Monash Medical Centre, Melbourne 3168, Australia; 3 Department of Paediatrics, Monash University, Melbourne 3168, Australia; 4 Monash Newborn, Monash Children’s Hospital, Melbourne 3168, Australia; 5 Australian and New Zealand Intensive Care Research Centre (ANZIC-RC), School of Public Health and Preventive Medicine, Melbourne 3004, Australia; ary.serpaneto@monash.edu; 6 Department of Critical Care, Melbourne Medical School, University of Melbourne, Austin Hospital, Melbourne 3084, Australia; 7 Department of Intensive Care, Austin Hospital, Melbourne 3084, Australia; 8 Department of Critical Care Medicine, Hospital Israelita Albert Einstein, Sao Paulo 05652-900, Brazil

**Keywords:** pregnancy, COVID-19, SARS-CoV-2, broccoli sprout, sulforaphane, clinical trial

## Abstract

Since its discovery in late 2019, the severe acute respiratory syndrome coronavirus 2 (SARS-CoV-2) has been estimated to be responsible for at least 769.3 million infections and over 6.95 million deaths. Despite significant global vaccination efforts, there are limited therapies that are considered safe and effective for use in the management of COVID-19 during pregnancy despite the common knowledge that pregnant patients have a much higher risk of adverse outcomes. A bioactive compound found in broccoli sprout—sulforaphane—is a potent inducer of phase-II detoxification enzymes promoting a series of potentially beneficial effects notably as an antioxidant, anti-inflammatory, and anti-viral. A pilot, double-blinded, placebo-controlled randomised trial is to be conducted in Melbourne, Australia, across both public and private hospital sectors. We will assess a commercially available broccoli sprout extract in pregnant women between 20^+0^ and 36^+0^ weeks gestation with SARS-CoV-2 infection to investigate (i) the duration of COVID-19 associated symptoms, (ii) maternal and neonatal outcomes, and (iii) biomarkers of infection and inflammation. We plan to enrol 60 outpatient women with COVID-19 irrespective of vaccination status diagnosed by PCR swab or RAT (rapid antigen test) within five days and randomised to 14 days of oral broccoli sprout extract (42 mg of sulforaphane daily) or identical microcrystalline cellulose placebo. The primary outcome of this pilot trial will be to assess the feasibility of conducting a larger trial investigating the duration (days) of COVID-19-associated symptoms using a broccoli sprout supplement for COVID-19-affected pregnancies. Pregnant patients remain an at-risk group for severe disease following infection with SARS-CoV-2 and currently unclear consequences for the offspring. Therefore, this study will assess feasibility of using a broccoli sprout supplement, whilst providing important safety data for the use of sulforaphane in pregnancy.

## 1. Introduction

The global pandemic for the severe acute respiratory syndrome coronavirus 2 (SARS-CoV-2) began in late 2019. SARS-CoV-2 is the cause of the coronavirus disease (COVID-19) and, as of 10 August 2023, has been responsible for over 769.3 million infections and at least 6.95 million deaths globally [1]. Pregnancy poses a significant risk for patients with COVID-19 with increased rates of premature birth, preeclampsia, stillbirth, and premature rupture of membranes; although, much of these data are informed from ancestral variants, such as the original Alpha and Delta variants [2]. Furthermore, pregnant women have increased risks of severe sequelae of COVID-19 in comparison to those who are not pregnant, having higher rates of admission to ICU, invasive ventilation, hospital admission, extra-corporeal membrane oxygenation (ECMO), and death [3]. More recent evidence suggests newer Omicron subvariants are associated with lower rates of preterm birth and maternal critical care admission compared to earlier strains [4]. Whilst vaccination appears to be safe and effective during pregnancy protecting against hospitalisation and severe disease caused by COVID-19 [5], vaccine hesitancy is more common in pregnant women [6,7,8]. Furthermore, pregnant patients were largely excluded from early COVID-19 pharmacological studies in both vaccination trials 97.8% (88/90) and treatment trials 70.7% (350/495) [9]. This results in a relative paucity of pregnancy-specific evidence for treatment options, leading to an initial reliance on safety data from non-pregnant population studies likely exacerbating the vaccine hesitancy observed during pregnancy [10,11].

A naturally occurring organosulphur and Isothiocyanate, sulforaphane (1-isothiocyanato-4-methylsulfinylbutane), is found within numerous cruciferous vegetables including kale and cabbage but is particularly concentrated within the aerial component of broccoli (*Brassica oleracea* var. *italica*) sprouts [12]. Sulforaphane is the product of metabolism of the glucosinolate glucurophanin via the enzyme myrosinase or via myrosinase-like activity from certain species of human gut microbiome bacteria [13]. Broccoli sprout extracts are easily produced and considerably cheaper than currently available monoclonal antibodies and anti-viral therapies, which is an important consideration when low- and middle-income countries continue to trail behind in COVID-19 vaccination rates [14]. Sulforaphane, present in broccoli sprout extracts, has been studied in over 70 completed or recruiting clinical trials since the late 1990s including malignancy, diabetes, gastroesophageal reflux, schizophrenia, asthma, and influenza [12]. Sulforaphane has anti-inflammatory, antioxidant, and anti-viral activity through its activity on the master gene regulator nuclear factor erythroid 2-related factor 2 (Nrf2) [15,16,17]. Nrf2 is a basic region leucine-zipper transcription factor predominantly known for its ability to bind and activate the antioxidant response element (ARE) of various genes to promote the expression of numerous phase II detoxification enzymes including glutathione S-transferase (GST), NADPH quinine oxidoreductase 1 (NQO-1), and heme-oxygenase 1 (HO-1) [18,19]. The evidence for an antioxidant effect from sulforaphane via its upregulation of Nrf2 has been summarised in a number of recent reviews [20,21,22]. Nrf2-driven genes have also shown suppression in lung biopsies from COVID-19-infected individuals, suggesting COVID-19 results in impaired cellular defence processes [23]. Rodrigues et al. [24] have also demonstrated the downregulation of Nrf2 in human umbilical vein endothelial cells (HUVECs) exposed to serum of COVID-19-infected individuals compared to healthy controls and a drastic reduction in nitric oxide (NO) production within 60 min of exposure. Early within the COVID-19 pandemic, Nrf2 was identified as a potential therapeutic target due to the theoretical beneficial effects on the oxidative stress and inflammation caused by the SARS-CoV-2 virus [17,25].

Our group has previously shown that sulforaphane has various beneficial effects including improving placental production of anti-angiogenic factors and protecting blood vessels from damage [26,27]. The commonly studied anti-angiogenic factor soluble fms-like tyrosine kinase 1 (sFlt1), commonly implicated in the pathogenesis of preeclampsia, was recently shown to have a significant association with COVID-19 disease severity and prognosis, highlighting the shared endothelial dysfunction between the two disease states [28]. It is this shared endothelial dysfunction across both COVID-19 and preeclampsia that has identified sulforaphane as a potential therapeutic in both the CO-Sprout study and another future trial with early onset preeclampsia [29,30]. Additionally, a pilot pharmacokinetic study has been performed using a broccoli sprout supplement analysing sulforaphane metabolite bioavailability in the circulation and the effects on systolic and diastolic blood pressure in women with gestational hypertension or preeclampsia, showing it was well tolerated with no adverse events seen [31]. In vitro, sulforaphane has demonstrated an ability to inhibit replication of SARS-CoV-2 [16] and in mouse models infected with multiple variants, displaying a synergistic effect when combined with the anti-viral therapy remdesivir [17]. Sulforaphane has also displayed beneficial endothelial stabilisation activity in both mouse microvascular endothelial cells [32] and HUVECs [33]. Finally, in vitro exposure of bronchial epithelial cells (IB3-1) to the SARS-CoV-2 spike protein and sulforaphane display reduced gene expression of the inflammatory interleukins IL-6 and IL-8, which are key components of the cytokine storm and potentially resultant acute respiratory distress syndrome (ARDS) [34].

Variants of concern including Omicron (B.1.1.529), its various subvariants BA.2-5, and newer recombinant variants such as XBB1.5 and XBF display an ability to escape the majority of existing SARS-CoV-2-neutralising antibodies [35]. This renders first-generation COVID-19 vaccines less effective against new infections compared with ancestral strains. More recently, short-term vaccine effectiveness of third or fourth vaccination with BNT162b2 mRNA (BioNTech, Mainz, Germany /Fosum-Pharma, Shanghai, China) or CoronaVac (Sinovac Life Sciences, Beijing, China) displayed rapid waning at 100 days after immunization when compared to 7 days further highlighting the need for strategies outside of vaccination alone, particularly for severe COVID-19 [36].

Sulforaphane has an established safety profile in both adult and paediatric human clinical trials [37] but with limited human pregnancy data outside of the previous work conducted by our group [31]; however, it has not yet been investigated in a large clinical trial against COVID-19. Animal pregnancy data indicate beneficial effects of sulforaphane on neonatal outcomes in various disease models including lipopolysaccharide-induced hepatic toxicity [38], foetal-growth-restriction-associated neurodevelopmental delays [39], chronic placental-insufficiency-induced foetal hypoxic-ischaemic encephalopathy [40], the induction of maternal immune activation with polyinosinic–polycytidylic acid [41] and hypoxic-ischaemic encephalopathy outcomes on piglets exposed to prolonged hypoxia [42]. COVID-19 in pregnancy has been implicated with maternal immune activation (MIA) with adverse foetal neurodevelopmental programming, highlighting the potential long-term effects on offspring born to pregnancies complicated by the disease [43].

The decision to complete this study as a pilot is primarily aimed at establishing feasibility, identifying challenges, and outlining future requirements for a larger randomised controlled trial. This is important, given the study design relies on home-visits with active COVID-19-infected pregnant patients in a large metropolitan area of Melbourne. Whilst establishing feasibility for future larger clinical trials, this study will simultaneously provide important initial safety data for use of sulforaphane during pregnancy.

## 2. Aims and Hypothesis

We hypothesise that a broccoli sprout supplement containing sulforaphane will reduce the duration of COVID-19-associated symptoms for pregnant women at 20^+0^ to 36^+0^ weeks gestation infected with SARS-CoV-2 and improve biochemical markers of inflammation. However, prior to establishing a larger clinical trial adequately powered to answer the clinical primary outcome, this study will investigate the unique challenges associated with an interventional outpatient study of COVID-19 pregnant participants investigating feasibility, recruitment, and patient identification strategies, patient acceptability, and work-flow logistics of patient and trial staff activities.

Aim 1. To assess the feasibility of conducting an outpatient study on COVID-19-infected pregnant patients requiring home-visits.

Aim 2. To assess if a broccoli sprout supplement containing sulforaphane can reduce COVID-19-associated symptom duration in pregnant women.

Aim 3. To investigate the effect of a broccoli sprout supplement on various biochemical markers of inflammation as well as pregnancy and neonatal outcomes.

## 3. Trial Design

**Figure 1:**
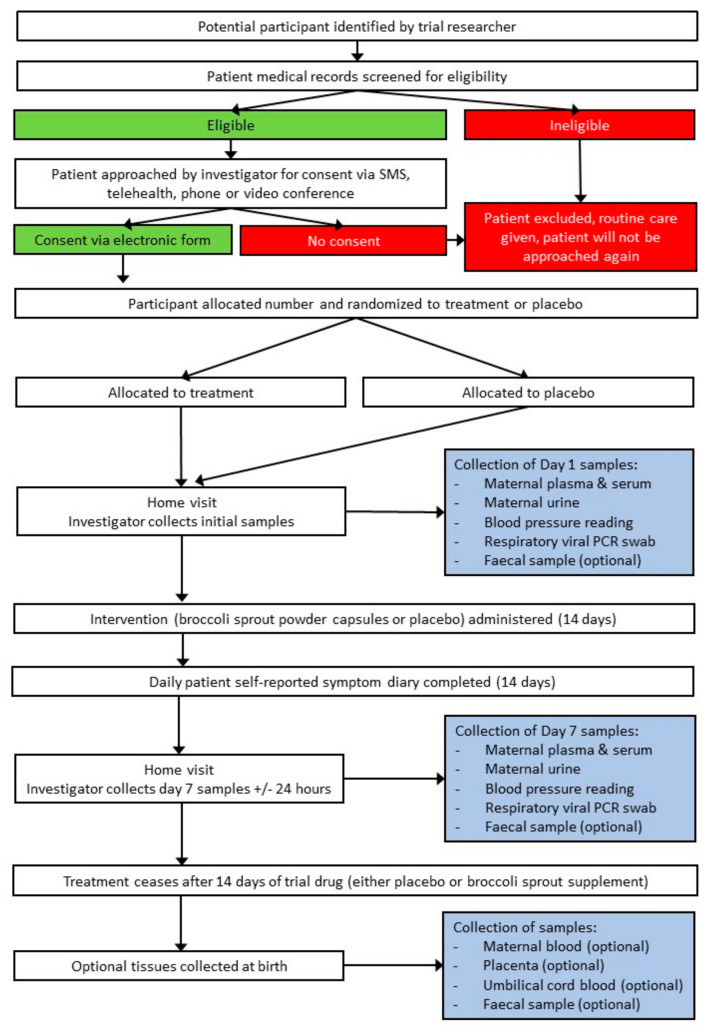
Patient procedures and study flowchart including participant identification, allocation to intervention, and home visits to collect biological samples (SMS—short messaging service; PCR—polymerase chain reaction).

This study is a pilot, double-blinded, parallel arm, randomised, placebo-controlled trial and has been summarised in a trial flow-chart below (Figure 1).

### 3.1. Sample Size

A convenience sample size has been set to a total *n* = 60 with 30 participants in each arm receiving either two broccoli sprout powder capsules GeneActive Formulation E (700 mg whole broccoli sprout powder) yielding sulforaphane (21 mg) twice daily or matching placebo capsule containing microcrystalline cellulose. Given that this is a pilot feasibility study, our convenience sample size is informed by predicted community COVID-19 infection rates during protocol design and predicted time until completion, allowing analysis of data to inform future work.

### 3.2. Participating Sites

Recruitment of pregnant women will occur at Monash Health Hospitals including Monash Medical Centre, Casey Hospital, Dandenong Hospital and Jessie McPherson Private Hospital, Victoria, Australia. These services cover a large catchment area of southeast Melbourne, Australia, caring for over 12,000 pregnant women and their babies each year. A centralised pilot trial steering committee will be established from the trial investigators for the oversight and management of the trial across the joint health networks.

### 3.3. Study Inclusion Criteria

A study participant must meet the following inclusion criteria to take part in this study:

- ≥18 years of age;
- Pregnant with singleton gestation at 20^+0^ to 36^+0^ weeks;
- Positive COVID-19 test either via viral PCR or RAT for SARS-CoV-2 within the preceding five days;
- Any vaccination status against SARS-CoV-2;
- Signs or symptoms of COVID-19 for ≤seven days before recruitment including but not limited to shortness of breath, anosmia, fevers, sore throat, headache, and myalgia;
- Able and willing to tolerate oral supplementation with a broccoli sprout extract for the full 14-day course;
- Able to understand the information provided in the participant information and consent form (PICF), and able to give written informed consent (with interpreter use as required).

### 3.4. Study Exclusion Criteria

A study participant will be deemed ineligible to take part in this study if they meet any of the following:

- Currently using a broccoli sprout extract or supplement;
- Contraindications to use of a broccoli sprout extract (e.g., intolerance of broccoli);
- Significant uncertainty of gestational age;
- Unwillingness or inability to follow the procedures outlined in the PICF;
- Mentally, cognitively, or legally incapacitated or ineligible to provide informed consent;
- Currently recruited in another clinical trial using a pharmaceutical, herbal, or nutritional intervention (such trial interventions would also include: multivitamins, minerals, antiviral, immunomodulatory or complementary and alternative medicines);
- Currently on an antibiotic, antiviral, or monoclonal antibody treatment related to acute illness.

### 3.5. Study Recruitment

Identification of potential trial participants will be predominantly via the local public health unit database of active COVID-19 cases from the community. Additionally, health staff and internally through the Monash Health Women’s and Newborn Department and Jessie McPherson obstetric services. Additionally, patients will be able to self-refer to the study team after testing positive to COVID-19 through the Monash Women’s and Newborn COVID-19 information webpage and through advertisement with flyers and informational posters in patient facing areas. Potential trial recruits will be contacted initially via Short Message Service (SMS) with links to the Monash Women’s website and patient information video outlining the trial. Trial staff will then contact patients via telehealth or phone. If interested in participation, patients will be provided the PICF via secure email (DocuSign, San Francisco, CA, USA). Electronic consent will be used where possible, allowing the study team to obtain consent remotely in an effort to reduce transmission of SARS-CoV-2 virus towards clinical trial staff. Additionally, participants can optionally consent to keeping remaining biological samples for further research studies via the PICF. Clinical trial staff will describe the trial, the voluntary nature of participation, and participation or not in the study will not in any way affect their routine clinical care. If patients consent to trial participation with signed electronic consent, they will be visited at home by the study team and provided study drug capsules, a study diary to record daily symptoms, and have collections of urine, blood, and respiratory viral PCR with an optional faecal sample.

### 3.6. Randomisation

Eligible patients will be randomised immediately after consenting to study enrolment. A permuted block randomisation with blocks of random size and stratification by study site and gestational age (20^+0^ to 27^+6^ and 28^+0^ to 36^+0^) will be used. Stratification by site has been utilised to minimise potential differences in population groups across different locations of study sites. Similarly, stratifying by gestational age has been undertaken in case of gestation specific risks from COVID-19 infection variably impacting on pregnancy complications such as preeclampsia, spontaneous pre-term birth, and foetal growth restriction. Patients will be allocated to the broccoli sprout supplement or placebo in a 1:1 ratio. A secure, centralised, web-based system for managing the study database (REDCap) will be used. This will allow for enrolment and randomised allocation to occur at any time of the day. A computer software program embedded into the REDCap system will generate a random allocation sequence for each patient, which will not be accessible to investigators, research coordinators, or study participants during the trial. PICF electronic copies will be uploaded into the password-protected secure REDCap database and other documents maintained on a password protected trial computer. Study participants will be randomised to either intervention. A clinical trial pharmacy has packed the investigational product and matched placebo capsules into sealed bottles with allocation blinded to study team and trial participants. The clinical trials pharmacy will keep a record of study participant identification number as well as treatment group allocation. At completion of the trial, unblinding of study drug interventions will occur by an independent data analyst not involved in data collection.

### 3.7. Interventions

Each trial participant will take two capsules twice daily (BD) of either 700 mg broccoli sprout powder (each capsule yielding 10.5 mg of sulforaphane), providing a total daily dose of 42 mg of sulforaphane (GeneActiv Formula E) or identical placebo-containing microcrystalline cellulose for 14 days. The study drug will be delivered to the trial participants’ homes along with a drug diary for recording capsule adherence, daily symptoms, and any adverse symptoms experienced during the trial from the intervention. Bottles will be collected after the 14-day intervention and the remaining capsules counted to measure adherence to the study protocol. In the event of severe disease requiring hospitalisation, patients will continue the allocated treatment remaining blinded unless concerns are raised by the treating medical team or patients are unable to continue oral therapies. Additional therapies following randomisation for treatment of COVID-19 including but not limited to monoclonal antibody or anti-viral medication will be documented and will not alter patient participation.

### 3.8. Study Limitations

The primary aim of this study is to serve as a pilot feasibility study for a larger future randomised controlled trial and as such will be underpowered to answer any clinical outcome related to the duration of COVID-19-associated symptoms. Several limitations, however, are noted in the study design for this trial. At current, the COVID-19 vaccination status of an individual does not preclude nor stratify them during the enrolment process. The study will report on vaccination rate, type of vaccination, number of vaccinations, and recency of vaccination in the reported outcomes. Furthermore, the primary outcome of this study is the duration of patient reported COVID-19-associated symptoms in a patient’s reported daily diary. It is appreciated that a more objective measure of SARS-CoV-2 viral load which is associated with COVID-19 symptom severity may be quantifiable by using a surrogate measure of viral load by reporting on cycle-threshold values (CT-values) or by daily PCR results across the duration of the study. Given that the primary outcome of the study is patient-reported duration of symptoms, this is what formed the primary study design of biological sample collection and the collection of samples prior to study intervention commencement (Day 1) and then a collection of further samples 7 days later. This will allow a review of CT-values between groups after randomization and 7 days of study intervention and, hence, an analysis of broccoli sprout extract on SARS-CoV-2 viral loads through a surrogate measure of the CT-value. Finally, as a nutritional supplement clinical trial, it may be possible to tightly regulate the nutritional status and dietary intake of study participants during the intervention period to limit dietary confounders in the study outcomes. This would only realistically be achieved by providing a 14-day complete nutritional diet to the study participant and additional household members, which has not been built into this study design. This study does not aim to control all dietary sources of sulforaphane-yielding vegetables and appreciate that possibly inflammatory diets such as those with a high-saturated-fat content will not be controlled for. Furthermore, the study will not be collecting patient nutritional intake though tools such as 24 h food-recall diaries, which we appreciate would provide a more complete overview of the study participants nutritional intake and, hence, address some potential confounders in the study such as alternative dietary sources of glucurophanin and, hence, sulforaphane.

## 4. Study Outcomes

As this trial is a pilot feasibility study, the primary goal is to establish future feasibility, challenges, and limitations of an outpatient COVID-19 study design in the South-East Melbourne region. COVID-19 and the public-health approach to managing the pandemic has shifted significantly in the last 12 months, particularly within Australia, with the reduction in morbidity and mortality seen with SARS-CoV-2 infection during Omicron-dominant periods. Identifying COVID-19-positive outpatient pregnant patients in an efficient and timely manner for recruitment to an interventional therapeutic study will be challenging in an era of “COVID-normal” and the reduction in patient-initiated queries to the health network. Additionally, this study will also assess the acceptability of a nutritional supplement for COVID-19 in the pregnant population living within South-East Melbourne. Finally, as this study will be the first to provide broccoli sprout powder yielding sulforaphane as a potential investigational therapeutic during pregnancy, this will also provide important initial maternal and neonatal safety information.

### 4.1. Primary Clinical Outcome

Our clinical trial primary outcome is the duration (days) of COVID-19-associated symptoms. These symptoms are to be reported in the patient self-reported study diary and include sore throat, hoarse voice, joint pain, muscle pain, nausea, vomiting, diarrhoea, irregular heartbeat, chills/shivers, abdominal pain, cough, shortness of breath, chest pain, fatigue, headache, runny nose, altered smell, and loss of smell. These symptoms will be reported as present/absent for each day of reporting without severity scales. Self-reporting up to day 14 of the study should be of sufficient duration, as reported from the ZOE COVID study finding Omicron-associated symptom duration to have a mean of 6.9 days and median of 5.0 days [44].

### 4.2. Secondary Clinical Outcomes

The secondary outcomes will be exploratory only and will broadly investigate the safety of broccoli sprout supplementation during pregnancy.

#### 4.2.1. Maternal and Birthing Outcomes

1. Birth outcomes including mode of delivery;
2. Obstetric complications including post-partum haemorrhage (PPH), pre-eclampsia, preterm pre-labour rupture of membranes (PPROM), and stillbirth;
3. Maternal death;
4. Unplanned hospital presentation within 28 days;
5. Admission to hospital for any reason with >24 h within 28 days;
6. Total duration of hospital admissions within 28 days;
7. Maternal biochemical, inflammatory, and anti-angiogenic markers;
8. Medication related adverse events;
9. Placental cellular and structural abnormalities (optional);
10. Gastrointestinal microbiome population species as measured through optional faecal microbiome testing (optional).

#### 4.2.2. Neonatal Outcomes

11. Birthweight and birthweight percentile;
12. COVID-19-positive polymerase chain reaction (PCR) swab in the neonate if testing performed as part of standard care;
13. Admission to neonatal unit and duration (special care nursery/neonatal intensive care unit);
14. Need for antibiotic therapy and duration of treatment;
15. Diagnosis of early-onset sepsis (EOS);
16. Diagnosis of late-onset sepsis;
17. Severe neonatal morbidity index (SNMI);
  - At least 1 of the following morbidities:
    - Bronchopulmonary dysplasia;
    - Hypoxic-ischemic encephalopathy;
    - Sepsis;
    - Anaemia requiring transfusion;
    - Patent ductus arteriosus;
    - Intraventricular haemorrhage;
    - Necrotizing enterocolitis;
    - Retinopathy of prematurity.
18. Severe perinatal morbidity and mortality index (SPMMI).
  a. Includes any of the indicators from SNMI and additionally:
    - Intrauterine foetal death;
    - Neonatal death;
    - Neonatal intensive care unit admission > 7 days.

#### 4.2.3. Maternal Respiratory Outcomes If Requiring Hospitalisation

19. Documented SpO_2_ < 94%;
20. Admission to intensive care unit (ICU);
21. Requirement for any oxygen therapy (maximal FiO_2_);
22. Requirement for non-invasive ventilation and/or high flow nasal cannula;
23. Requirement for mechanical ventilation;
24. Requirement for ECMO;
25. Diagnosis of maternal acute respiratory distress syndrome (ARDS) as defined by the European Society of Intensive Care Medicine as new or worsening respiratory symptoms that includes a combination of acute hypoxaemia (PaO_2_/FiO_2_ ≤ 300 mm Hg) in a ventilated patient with positive end-expiratory pressure (PEEP) of at least 5 cm H_2_ and bilateral opacities not fully explained by heart failure or volume overload that occurs within 7 days of a clinical insult (37);
26. Maximal disease severity of COVID-19 as defined by the Society for Maternal Foetal Medicine (SMFM) [45].

Electronic medical records will be utilised to record maternal and neonatal outcomes, as well as to collect maternal demographics including body mass index (BMI), ethnicity, drug and alcohol use, smoking status, and maternal comorbidities.

## 5. Sample Collection and Storage

Biological specimens collected on days 1 and 7 of the trial during planned home visits include blood, urine, oro-pharyngeal nasal PCR swab, and an optional faecal sample. At the time of birth, optional maternal and umbilical cord blood, placenta, and faecal sample will be collected. Participant maternal peripheral blood and umbilical cord blood will be placed into both ethylenediaminetetraacetic acid (EDTA) and clot-activating blood tubes for both plasma and serum collection. Biochemical and inflammatory bloodwork collected from trial participants will be analysed through Monash Health Pathology and include full blood examination (FBE), electrolytes, urea and creatinine (EUC), liver function testing (LFTs), C-reactive peptide (CRP), ferritin and coagulation studies. Once blood has been collected, EDTA tubes will be placed on ice, the others will remain at room temperature for transport to hospital pathology for routine processing. Additional EDTA and silica (clot-activator) tubes will be taken to the laboratory and centrifuged at 1200× *g* for ten minutes and both plasma and serum stored at −80 °C. The plasma and serum will be analysed for angiogenic and antiangiogenic markers including sFlt-1, sEng (soluble endoglin), and PlGF (placental growth factor). Urine samples will be collected in sterile specimen containers and then transferred on ice to the laboratory and stored in collection tubes at −80 °C. Faecal specimen collection will be undertaken using sterile self-contained faecal sample collection kits (ABC, Protocult™) and stored at −80 °C. Faecal samples have been collected for possible future analysis of microbiome alpha and beta diversity following exposure to broccoli sprout or placebo. Respiratory viral PCR swabs (Kangjian Virus Collection and Preservation System) for SARS-CoV-2 genotyping and for the calculation of CT (cycle threshold) values as a surrogate marker of viral load will also be collected. Placental samples collected at the time of birth will be transported on ice to the laboratory. Placental samples will include four 1 cm^3^ sections of placenta that will have the maternal and foetal sides removed and washed in phosphate-buffered saline (PBS). Samples will then be snap-frozen in liquid nitrogen and prior to storage at −80 °C. Full sections of the placenta both peripheral and central as well as sections of umbilical cord will be preserved in 10% neutral buffered formalin (NBF) and then fixed in paraffin wax. The placenta will also be sent for formal histology reporting by the site’s anatomical pathology department.

## 6. Proposed Analysis

In addition to primarily investigating feasibility for a larger broccoli sprout supplement clinical trial, we will also investigate the ability of sulforaphane to reduce COVID-19-associated symptom duration for pregnant patients with COVID-19. This will be reported as mean duration (days) of symptoms of participants between the investigational product (broccoli sprout powder) and placebo to a maximum of 14 days of symptom recording. Data will be collated and analysed according to the intention-to-treat principle. The primary outcome analysis will be performed using a Fine–Gray competing risk model with death before the event (resolution of symptoms) treated as a competing risk. Appreciably, with the reduction in severe morbidity and mortality, it is unlikely that death before the event (resolution of COVID-19-associated symptoms) will occur. Regardless, in the unfortunate setting of a new variant associated with a more severe disease phenotype, the Fine–Gray model will provide a more accurate method for the analysis when compared to other statistical models, as it will accommodate the competing risk of death in the situation it occurs. Additionally, results will be reported as sub-distribution hazard ratios (SHR) and 95% confidence intervals. In the competing risk models, subjects who experience death remain in the risk set and the SHR denotes the instantaneous rate of the event of interest in subjects who are still alive (i.e., who have not yet experienced either death or the event of interest) and also those who have died. *p*-values of <0.05 will be the cut-off for statistically significance. We do not plan to undertake any interim analysis for this pilot trial.

## 7. Trial Related Matters

### 7.1. Adverse Events and Study Insurance

Sulforaphane, provided as a broccoli sprout extract, is a naturally occurring bioactive compound that has been investigated in many human clinical trials [37]. Based on animal studies and our previous work, we do not expect to encounter any serious adverse outcomes due to the intervention. However, broccoli sprout supplementation has not been studied in a large human pregnant population. Whilst there have been no serious adverse outcomes reported in relation to use of broccoli sprout supplement, it is possible that unforeseen and unexpected adverse outcomes may occur during the clinical trial. Study trial participants will have access to a 24 h phone number that will be held by the chief principal investigator or delegated trial researcher and are advised to call if any concerns arise during the study duration. Furthermore, routine clinical care for all participants will continue as normal and study participants will be encouraged to call the Pregnancy Assessment Unit (PAU) at their corresponding hospital at any stage for review by midwifery or medical staff as needed. In addition, routine biochemistry will be performed as part of the study to identify any adverse effects unexpected.

In the event of adverse events occurring, the assessment and reporting of such events will occur as outlined by the Sponsor (Monash Health) and the National Health and Medical Research Council (NHMRC). Adverse events and serious adverse events (SAE) will be recorded and reported in detail within the participants’ electronic medical record and submitted to Monash Health and Monash Health Human Research and Ethics Committee (HREC) for further assessment. Study participant data will be collected electronically and stored securely on the web-based REDCap trial database allowing retrieval of information for audit or review as required. The Sponsor, Monash Health, is responsible for the provision of insurance for the trial through the Victorian Managed Insurance Agency (VMIA).

### 7.2. Trial Discontinuation or Modification

The sponsor Monash Health has had no ultimate authority over the design of the clinical trial, trial management, patient recruitment, data or sample collection, analysis or the interpretation of data, or the writing of the manuscript for publication. The principal investigators NJF, KRP, DLR, and SAM will form a trial steering committee to oversee trial management. Any amendments to trial protocol will be registered with ANZCTR and submitted to the publishing journal. As this is a pilot phase I study, there will not be a data safety management board (DSMB) convened, given the exploratory nature of the outcomes. Instead, a trial group will oversee the trial and review any safety reports and overall quality of the data including participant retention and protocol adherence.

Prematurely, permanent, or temporary ceasing of recruitment will occur if the chief principal investigator or the sponsor (Monash Health) believes there are issues relating to any, or all of:

- Participant welfare and safety;
- The conduct of the trial in accordance with the Monash Health HREC-approved protocol;
- A failure to adhere to Monash Health Research Directorate and HREC conditions of approval;
- A recommendation from the trial group that the trial should cease completely due to safety concerns.

The trial will conclude as per study protocol when:

- The researchers have recruited, as per protocol, all trial participants (*n* = 60);
- Data collection and entry is complete, with subsequent independent verification;
- Database lock has occurred;
- Completed data analysis;
- The trial has been conducted as per Monash Health HREC approval and the necessary reporting (e.g., Annual Report, SAE) has been completed and acknowledged.

The trial cannot be modified after recruitment has commenced, unless approved by HREC.

### 7.3. Unblinding

The trial can only be unblinded in the following circumstances:

- To inform clinical management or under conditions of an SAE where disclosure of the intervention is necessary;
- At the conclusion of the trial to determine the effect of the intervention.

### 7.4. Ethics and Dissemination

This trial was reviewed and approved (RES-21-0000-708A) by the Monash Health Human Research Ethics Committee (HREC) with registration on the Australian and New Zealand Clinical Trials Registry (ANZCTR:12622000173796) prior to commencement. This trial will be conducted in compliance with local and NHMRC guidelines. The trial will occur as stipulated by the clinical trial protocol and amendments or alterations in the clinical trial protocol will only occur after written approval by the Monash Health HREC unless there is an apparent and immediate patient safety risk. Study participants will be able to optionally request to be informed of their allocation and study outcomes at trial completion. Clinical trial outcomes will be disseminated locally, nationally, and internationally at meetings and conferences in peer-reviewed journals.

## 8. Discussion

The CO-Sprout study is the first randomised controlled trial to investigate a broccoli sprout extract as a potential therapeutic in pregnancy targeting excessive inflammation and oxidative stress via the active ingredient sulforaphane. COVID-19 in pregnancy results in a state of inflammation and oxidative stress despite largely limiting vertical transmission to the foetus [46]. Severe COVID-19 is associated with an increased risk of adverse pregnancy outcomes, including preeclampsia and stillbirth during pregnancy. Furthermore, the infection with SARS-CoV-2 may result in maternal immune activation and resultant foetal neuroinflammation. Foetal neuroinflammation is a well-established risk factor for neurobehavioral adversity in children of affected pregnancies [43,47]. Similarly, excessive levels of oxidative stress, inflammation, and immune dysregulation many disorders of pregnancy including preeclampsia, foetal growth restriction, and diabetes [48]. The use of a natural broccoli sprout supplement yielding sulforaphane in the CO-Sprout study provided an opportunity to explore a novel therapy for disorders of oxidative stress and inflammation, such as COVID-19 in this high-risk pregnant population.

Sulforaphane has potent antioxidant and anti-inflammatory effects, implying it may have other potential therapeutic indications throughout pregnancy, both stabilising the maternal system but also through mitigation of some of the harmful effects to the developing foetus and placenta as seen with maternal immune activation. CO-Sprout will investigate important safety questions for use of sulforaphane in pregnancy, appreciating that this study will be underpowered for most rare safety outcomes. Further investigational studies will be required, particularly to investigate the short and long-term infant outcomes for mothers exposed to sulforaphane in pregnancy. However, given our previous work on broccoli sprout supplementation in pregnant individuals and the pre-existing evidence base, both laboratory and animal, we do not envisage any adverse outcomes for either mother or baby from exposure to broccoli sprout powder. Regardless, this study will collect both maternal and neonatal outcomes to further establish the evidence base and inform future trials and studies.

## Data Availability

Upon completion and publication of the trial results, de-identified trial data will be made available to others upon reasonable request. Such requests should be made to: Neville Fields at neville.fields1@monash.edu.
